# Collective transient ratchet transport induced by many elastically interacting particles

**DOI:** 10.1038/s41598-021-95654-8

**Published:** 2021-08-10

**Authors:** Cesar Manchein, Tulio M. de Oliveira, Rafael M. da Silva, Marcus W. Beims

**Affiliations:** 1 Departamento de Física, Universidade do Estado de Santa Catarina, Joinville, SC 89219-710 Brazil; 2 Departamento de Física, Universidade Federal do Paraná, Curitiba, PR 81531-980 Brazil; 3 Max Planck Institute for the Physics of Complex Systems, Nöthnitzer Str. 38, 01187 Dresden, Germany

**Keywords:** Nonlinear phenomena, Statistical physics, thermodynamics and nonlinear dynamics

## Abstract

Several dynamical systems in nature can be maintained out-of-equilibrium, either through mutual interaction of particles or by external fields. The particle’s transport and the transient dynamics are landmarking of such systems. While single ratchet systems are genuine candidates to describe unbiased transport, we demonstrate here that coupled ratchets exhibit *collective transient ratchet transport*. Extensive numerical simulations for up to $N=1024$ elastically interacting ratchets establish the generation of large transient ratchet currents (RCs). The lifetimes of the transient RCs increase with *N* and decrease with the coupling strength between the ratchets. We demonstrate one peculiar case having a coupling-induced transient RC through the asymmetric destruction of attractors. Results suggest that physical devices built with coupled ratchet systems should present large collective transient transport of particles, whose technological applications are undoubtedly appealing and feasible.

## Introduction

Besides some key developments related to fluctuations theorems^1^, nowadays the understanding of the fundamentals of nonequilibrium physics is still incomplete. It has been shown^2,3^ that out-of-equilibrium systems may remain in a thermodynamic state, which is the continuation of the equilibrium state under weak nonequilibrium constraints. Beyond some arbitrary threshold, the thermodynamic state becomes unstable and new states emerge by bifurcation with spatial or temporal inhomogeneities^4^. Consequently, such out-of-equilibrium systems can present anomalous (paradoxical) behaviors since the laws of equilibrium thermodynamics no longer possess validity: In special, we mention the anomalous transport phenomena observed in out-of-equilibrium systems in different problems. This scenario can be illustrated in a biological system, where the anomalous diffusion of the energy landscape in human chromosomes takes place^5^, in classical systems^6–8^, where Brownian particles moving in a system driven by thermal fluctuation and external forces that can exhibit anomalous transports, in collective motion and chaotic states^9^ or turbulent state^10^, in active particles^11^, in living cells^12,13^, in cold atoms^14–16^ and in quantum systems^17–22^, to mention a few.

Unveiling the main properties which produce the transport phenomena is a central issue in numerous problems in physics, and it is, undoubtedly, a challenge in spatially extended systems away from equilibrium. In coupled many-body quasi-integrable systems, the phase-space diffusion time is much longer than the Lyapunov time of the underlying chaotic dynamics. Consequently, relaxation processes leading to thermal equilibrium through mutual interaction between the bodies involve slow dynamics and a long transient motion may appear for intermediate times. Actually, for finite systems with *N* degrees of freedom, the time to reach the equilibrium state may increase without bounds as *N* increases and, subsequently, the time of the transient dynamics can be prolonged proportionally. In realistic situations and some numerical simulations, for larger values of *N* such enormous times can never be reached, and considering long transients become essential to describe the transport properties of the physical system.

Of particular interest in this context is the *transient property of the net transport* of many interaction particles due to the ratchet effect and the collective behavior. Ratchet transport is a directional transport of many particles in spatially periodic media, which results in the rectification of an external net-zero force applied to the system. After the theoretical contributions of Gabriel Lippmann, Marian Smoluchowski, and Richard Feynman^23^, the idea of a ratchet device was first adapted to biological circumstances and applied to study the transport of proteins within cells using molecular motors^24–26^. Connected to this, due to the influence of the thermal fluctuations on the movement of macromolecules, the dynamics described by a protein can be considered equal to the dynamics of an overdamped Brownian particle, in which the inertial term is neglected, and the forces generate speeds instead of accelerations^27^. With the advances of the research in this field, another phenomenology takes place in which thermal fluctuations became unnecessary to generate directional transport. Therefore, it is possible to obtain deterministic ratchet systems with the following indispensable requirements: the existence of an inertial term, dissipation, and a periodic potential capable of break the spatial symmetry of the system^28,29^. With the inertial term, the number of first-order differential equations necessary to describe the system increases, and it is possible to find a chaotic dynamic for the particle^29^.

While the literature regarding the transport of single ratchet particles systems is huge^27,30–39^, to mention a few, not much has been done concerning the transport in coupled ratchet systems where the collective dynamics arise. We acknowledge the case of an elastically coupled lattice of particles in a periodically flashing ratchet potential^40,41^, where the transport efficiency improves when the coupling strength overcomes a threshold. The case of optimal transport of two elastically interacting particles was analyzed recently in the parameter space^42^, and also shown that hydrodynamic interactions between Brownian particles influences the performance of a fluctuating ratchet^43^. Completely absent, as far we know, are studies related to transient ratchet transport properties due to the mutual interaction of many particles.

In the present work, we describe the transient Ratchet Currents (RCs) of $N=64,~128,~256,~512$, and 1024 elastically interacting particles, each subjected to a ratchet system. Using extensive numerical simulations, we show that the transient lifetime ($\tau$) of the RC increases with *N* as a power-law. As expected, for a fixed *N*, the transient RC disappears for asymptotic times. However, in the limit $N\rightarrow \infty$, the RC’s transient time follows $\tau \rightarrow \infty$, and the asymptotic current converges to the current from the uncoupled case. The observed transient RCs are a consequence of each particle’s current separately, which for asymptotic times disappear due to the coupling which induces the hyperchaotic dynamics. We also demonstrate one case having a coupling-induced transient RC through the asymmetric destruction of attractors. From the statistical point of view, as confirmed by the Lyapunov spectrum, the multiple attractors on the microscopic level generate the macroscopic transient RC. Furthermore, for larger couplings ($\beta$) between the ratchets, the transient RCs diminish, also as a power-law.

## Results

### The uncoupled case

**Figure 1:**
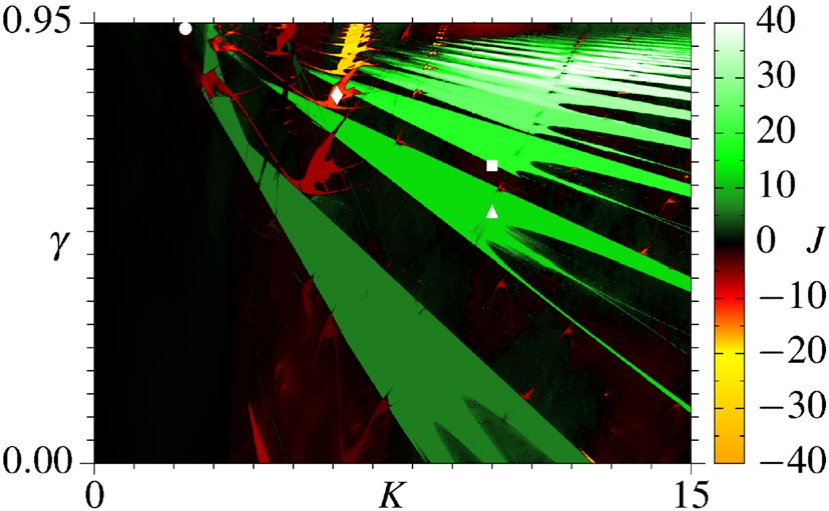
Ratchet current *J* in the parameter space of the uncoupled case ($\beta = 0$) for the coupled ratchets model (1). The parameters are in the intervals $\gamma \in \left[ 0.0,~0.95 \right]$ and $K \in \left[ 0.0, ~15.0 \right]$ and the iteration time is $10^{6}$ for 128 initial conditions.

For later reference, Fig. 1 displays the RC (see color bar) in the parameter space ($K,\gamma$) for the uncoupled case $\beta =0$ from Eq. (1). Black colors refer to close to zero RCs, green to white to increasing positive RCs, and red, yellow to orange to increasing negative RCs. It has been shown previously^44^ that optimal and efficient RCs occur when parameters are chosen inside Isoperiodic Stable Structures (ISSs), which are the green, red and yellow structures in Fig. 1 having well-defined borders. In such cases, the particle dynamics is periodic and Lyapunov stable. The black background in Fig. 1, related to nearly zero RCs, belongs to those parameter’s combination for which the underline dynamics is chaotic. For more details, we refer the readers to original work regarding RCs in parameter space^44^. The symbols $\bullet$, $\blacklozenge$, $\blacksquare$ and $\blacktriangle$ in Fig. 1 are related to pairs of parameters chosen in different regimes, with the respective values $(K,\gamma )=(2.3,~0.94)$, (6.1,  0.80), (10,  0.65), and (10,  0.55). These are the parameter pairs for which the coupling between the ratchets is explored in the present work. At point $\blacktriangle$ we have a stable period-1 dynamics and the RC is $J=4\pi \sim 12.564$. The pair indicated by $\blacksquare$ is at the borderline between chaotic motion with $J \sim 0$ and the stable period-1 motion with $J=6\pi \sim 18.723$. At $\blacklozenge$ we have a stable period-2 dynamics with $J=-4\pi \sim -12.546$. Finally, the point $\bullet$ belongs to a chaotic region with $J \sim -1.521$, for which the underline dynamics contains special properties that lead to the temperature enhanced RC, as demonstrated almost a decade ago^45^. Thus, the above points are representative of a diversity of dynamics observed in the uncoupled case.

### Coupled ratchet currents

**Figure 2:**
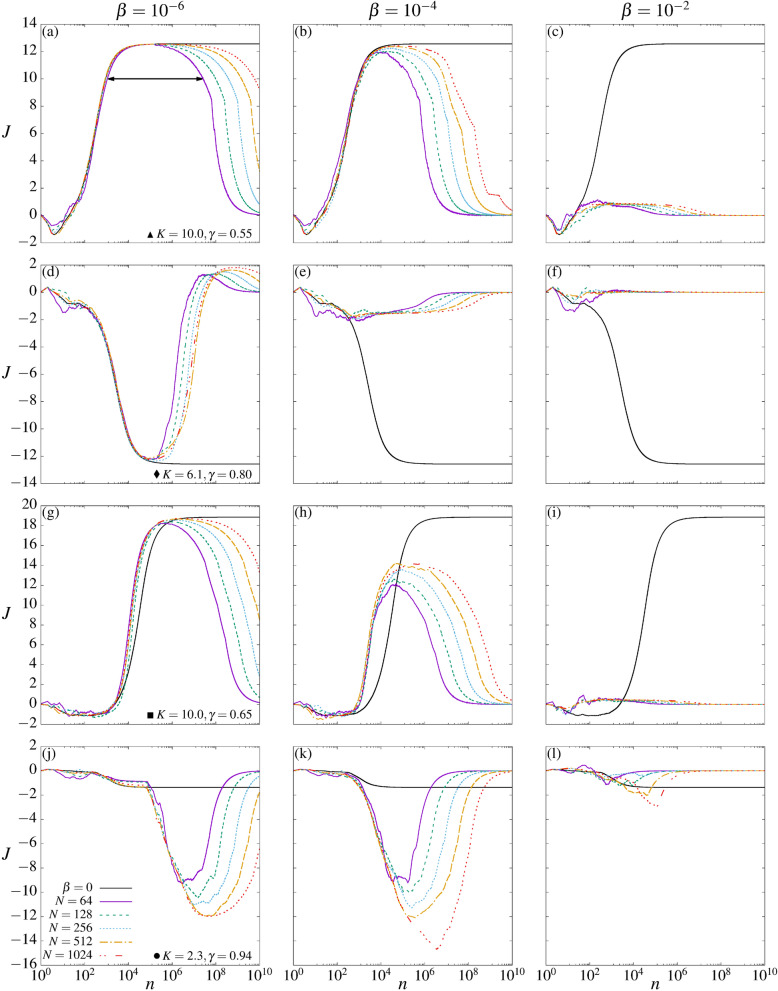
Curves for the RC *J* as function of iteration time *n* and for a distinct number *N* of coupled systems. The pair of parameters are $K=10.0, \gamma =0.55$ for (**a**–**c**), $K=6.1, \gamma =0.80$ for (**d**–**f**), $K=10.0, \gamma =0.65$ for (**g**–**i**), and $K=2.3, \gamma =0.94$ for (**j**–**l**). The black continuous curves are the *J* for the corresponding parameters in the uncoupled cases, for 1024 initial condition. The arrow in (**a**) exemplifies how the lifetime $\tau$ is determined numerically.

Figure 2 displays the curves for the RC *J* as a function of the iteration time *n* and for different values of *N*, the number of coupled maps. The coupling strength increases from the left to the right columns, namely $\beta =10^{-6},~10^{-4}$, and $10^{-2}$, respectively. Figures on the same line correspond to the dynamics of the specific parameters from Fig. 1, denoted by $\blacktriangle$ (Fig. 2a–c), $\blacklozenge$ (Fig. 2d–f), $\blacksquare$ (Fig. 2g–i), and $\bullet$ (Fig. 2j–l) (see Figure caption). The black curves in Fig. 2 are the RCs from the uncoupled case, whose asymptotic values are plotted in Fig. 1 for the specific points mentioned above. When the coupling is turned on, RCs are generated in all cases with $\beta \le 10^{-4}$ as a consequence of the existence of a collective behavior. These RCs are transient since they disappear for $n\rightarrow \infty$.

Figure 2a–c display the RC for the period-1 dynamics found at the $\blacktriangle$ point from Fig. 1. The curves obtained by using $N=64\,k$ (for $k=1,2,4,8,16$) coupled maps are distinguished by different colors (see labels). Note that in Fig. 2a, for $\beta =10^{-6}$, all curves start with the RC close to zero until they reach a maximum $J\sim 4\pi$ around times $n\sim 10^5$. These maxima are very close to the asymptotic limit of the uncoupled case. Furthermore, they are transient and for larger times converge to zero. For increasing values on *N*, the times to converge to zero increase. Essentially, the same behavior is observed in Fig. 2b for $\beta =10^{-4}$, but the transient maxima occur for smaller times, namely $n\sim 10^4$. For larger couplings ($\beta =10^{-2}$), we see in Fig. 2c that the above transient property of the RC disappears and remains around zero for all times.

Figure 2d–f display the RC for the period-2 dynamics found at the $\blacklozenge$ point from Fig. 1 for the same values of $\beta$ used in Fig. 2a–c. After an initial short time interval presenting $J\sim 0$, the RCs in Fig. 2d reach a transient minimum close to $J\sim -4\pi$ at times $n\sim 10^5$. These minima are again close to the asymptotic limit of the uncoupled case. After this time, the RCs reach a transient maximum close to $J\sim 1$ and finally converge to zero. As seen above, for increasing values on *N*, the times to converge to zero increase. For coupling $\beta =10^{-4}$ the transient minima at $J \sim -2$ are smeared out and converge to zero after that (see Fig. 2e). Increasing the coupling to $\beta =10^{-2}$ the relevant transient RCs disappeared. Basically, the same behavior is observed in Fig. 2g–i for the parameters relative to the $\blacksquare$ in Fig. 1. Here the transient maxima come close to $J \sim 6\pi$ for $\beta =10^{-6}$ and for times $n \sim 10^6$. However, for increasing couplings, the times for the transient maxima decrease.

Finally, we present the point $\bullet$ from Fig. 1. The corresponding RCs are observed in Fig. 2j–l. The main difference from the other cases is that the RC from the uncoupled case is close to zero, and we do not expect any larger transient RC for intermediate times. However, as seen in Fig. 2j, k, relevant RCs $-14\lesssim J \lesssim -8$ occur for times $10^6\lesssim n\lesssim 10^8$, depending on *N*. As *N* increases, the transient minima decrease, approaching significant negative values of the RCs. Thus, a coupling-induced transient RC is generated. For larger values of the coupling, these effects tend to vanish, as seen in Fig. 2l.

**Figure 3:**
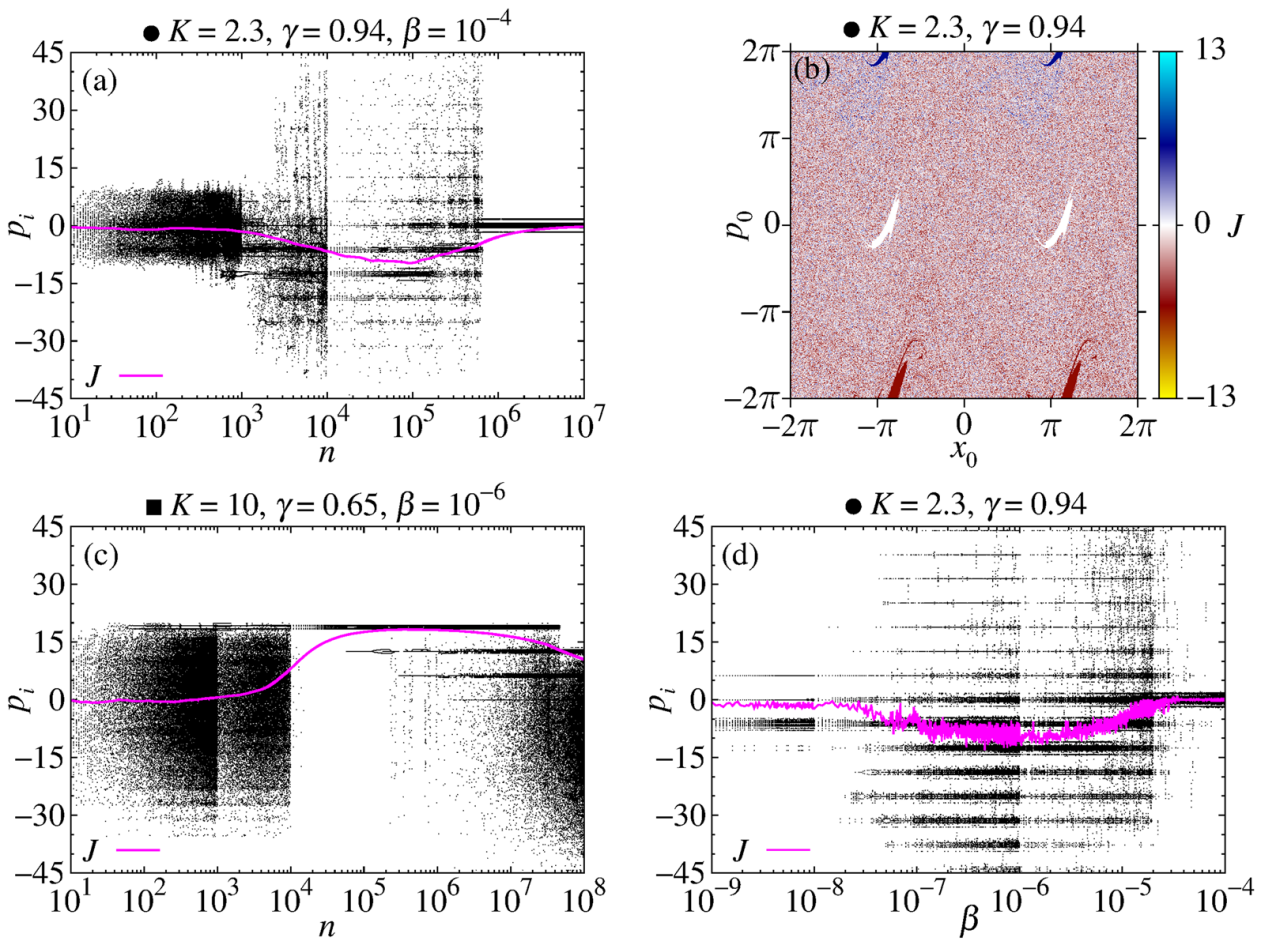
(**a**) and (**c**) We plot $p_i \times n$ for the parametric combinations indicated by $\bullet$ and $\blacksquare$ in Fig. 1, respectively. (**d**) Displays $p_i \times \beta$ with $n_{\mathrm{trans}}=2\times 10^6$ and $n=10^6$ for the point $\bullet$. The pink curves plotted in these diagrams represent the respective RC. (**b**) It is plotted the basin of attraction for only one ratchet map, for $K=2.3$, $\gamma =0.94$, and $n=10^5$.

In order to unveil the reasons responsible for the activation of the transient RCs, we plot in Fig. 3a, using $\beta =10^{-4}$ and $N=64$, the quantity $p_i \times n$ for the parametric combination indicated by $\bullet$ in Fig. 1. Initial conditions are equally distributed between $-2\pi$ and $2\pi$ for the pairs ($x_i,p_i$). The RC *J* as function of time *n* is plotted in pink color. For times $n\lesssim 10^3$, $p_i$ remains symmetrically distributed around zero, leading to $J\approx 0$. For times $10^3\lesssim n\lesssim 10^6$, $p_i$ values diffuse asymmetrically having a larger density of points for $p_i<0$, inducing $J<0$. After that, all maps have $p_i\approx 0$. In Fig. 3b, we plot the basin of attraction indicating in colors the RC for each of the $10^3 \times 10^3$ initial conditions $(x_0,p_0)$ considering only one ratchet map. Note that, in this case, the attractor around $p = 2\pi$ is smaller than the attractor around $p = -2\pi$, which induces negative RCs for the uncoupled case and the negative transient RCs for the coupled case. These attractors exist also for *p* values multiple of $2\pi$. For the coupled case, the ICs may approach these attractors, but only after $10^3$ iterations the asymmetry becomes relevant, leading to the transient negative *J*, as seen in Fig. 3a. In Fig. 3c we consider the same quantity but for the parametric combination indicated by $\blacksquare$ in Fig. 1. Interesting to observe in this case is that, already for the first iterations, the $p_i$ values assume large values which exceed the range of initial conditions of $-2\pi$ to $2\pi$. In other words, the coupling between the ratchets allows particles to visit temporarily attractors with higher values of *p*. The asymmetry of the attractors becomes relevant for $n\gtrsim 10^4$, leading to the transient $J\approx 6\pi$, which for $n\gtrsim 10^8$ tend to vanish. Figure 3d displays the values of $p_i$ as a function of $\beta$ for $K=2.3$ and $\gamma =0.94$. We see that for $10^{-7}\lesssim \beta \lesssim 10^{-5}$ many transient attractors are reached through diffusion in phase space generating a strong negative RC. Therefore, we obtain a *coupling-induced transient* RC.

### Ratchet current’s lifetime

**Figure 4:**
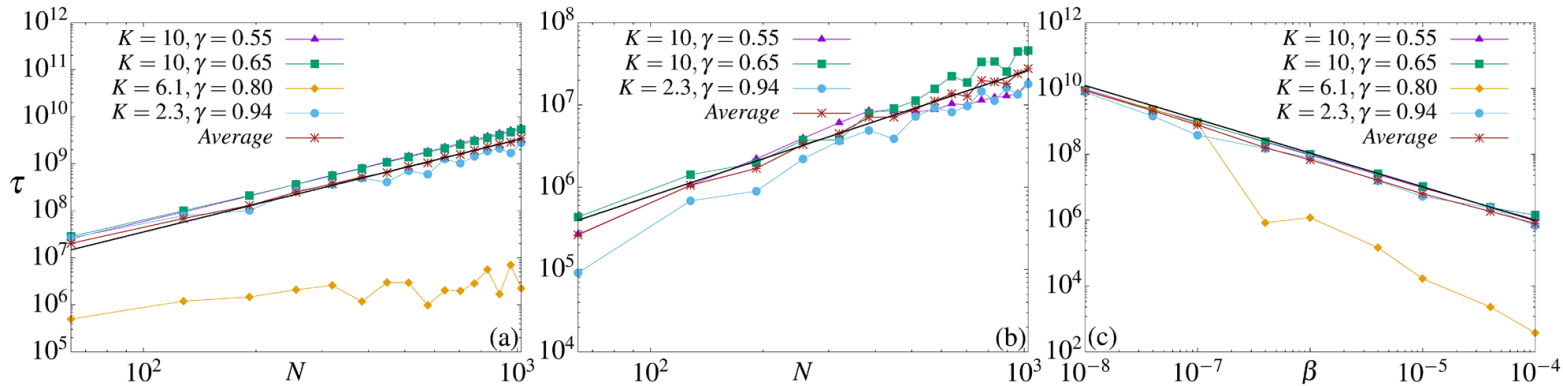
RC’s lifetime $\tau$ as a function of *N* for (**a**) $\beta =10^{-6}$ and (**b**) $\beta =10^{-4}$. The black lines are power-law functions $\tau (N)\approx\alpha N^{\chi }$ that adjust the brown curves obtained through the average computed over all studied cases (excluding the case of $(K,\gamma )=(6.1,0.80)$ in (**b**) ). (**c**) The RC’s lifetime is plotted as a function of $\beta$ for $N=128$. The black line decays with $\tau {(\beta )} \approx {\nu }\beta ^{-\mu }$, function which adjusts the brown average curve.

The properties of the collective transient RCs from Fig. 2 can be analyzed for different values of *N* and $\beta$. Let $\tau$ be the RC’s lifetime, defined as the time for which |*J*| remains equal or greater than $80\%$ of the maximal absolute value of RC found along the iteration time for a specific value of *N* (In fact, for small couplings, the decay exponents for the lifetime, presented next, are similar using thresholds between $\sim 30\%$ and $\sim 90\%$). See the arrow in Fig. 2a for one example. Figure 4a,b show the log–log plot of $\tau$ as a function of *N* for $\beta =10^{-6}$ and $\beta =10^{-4}$, respectively. Parameter pairs ($K,\gamma$) are the same as before. The increasing of the current’s lifetime for all cases obeys the power-law $\tau (N)\approx \alpha ~N^{ \chi }$ with the same exponents for a fixed $\beta$, namely $\chi =1.96178 ( \pm 0.03888)$ in Fig. 4a and $\chi =1.51159 ( \pm 0.1006)$ in Fig. 4b. Figure 4c displays the log–log plot of $\tau$ as a function of $\beta$ for $N=128$ and distinct values of ($K,\gamma$), according the symbols from Fig. 1. The black line is the power-law regression fit of the brown averaged curve which decays with exponent $\mu =1.02609 \pm 0.00068$.

**Figure 5:**
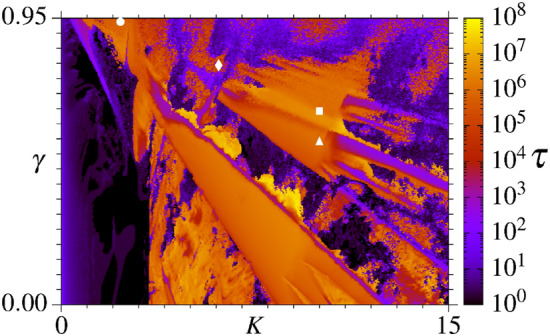
RC’s lifetime $\tau$ in colors according to the color bar (in logarithm scale) in the parameter space for $\beta =10^{-4}$ and $N=128$ elastically interacting particles in system (Eq. 1).

For real applications, the collective RC transient effect is essential. For some tiny coupling strengths and a huge number of particles, finite RC’s lifetimes $\tau$ could be too large to be observed in experiments, and the transient features turn out to be interpreted as the asymptotic behavior. The values of $\tau$ change depending on the underline dynamics. To give a more general view of this, Fig. 5 displays the RC’s lifetime $\tau$ (see color bar) for $\beta =10^{-4}$ and $N=128$ for the same parametric interval from Fig. 1. We see a rich alternation of colors and, when compared to Fig. 1, allows us to make the following statement: the general behavior is that most of the large values of $\tau$ (orange and yellow regions) occur for parameters relative to a periodic and Lyapunov stable motion. Some exceptions occur around to the largest green ISS from Fig. 1.

### Lyapunov spectrum

**Figure 6:**
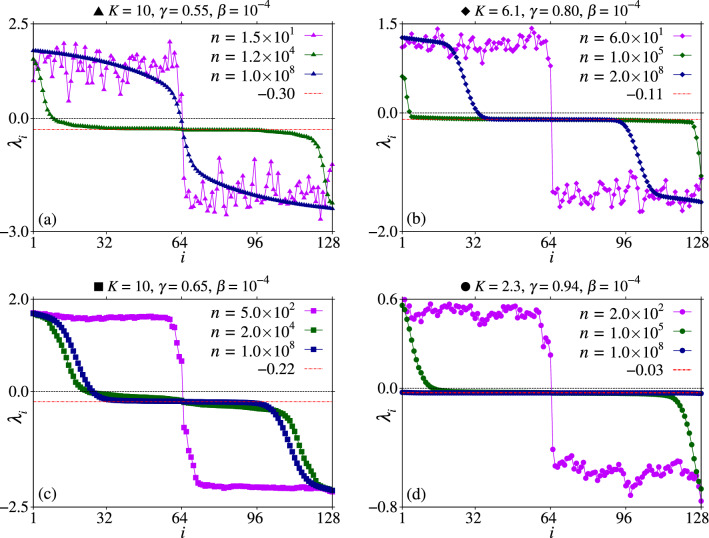
The spectrum of FTLEs for $N=64$ and $\beta =10^{-4}$ by using the same parametric combinations from Figs. 2 and 4 and different values for the iteration time *n*. The symbols used correspond to the symbols from Fig. 1. The red dashed-dotted lines indicate the value of the FTLE $\lambda$ obtained for the uncoupled map.

Figure 6 shows the spectrum of finite-time Lyapunov exponents (FTLEs) $\lambda _i$ for $\beta =10^{-4}$, $N=64$, and three distinct times: for short times (magenta) for which the RC is close to zero, for intermediate times (green) with the large transient RC, and for considerable times (dark blue) for which the RC disappears. The red dashed-dotted lines indicated the corresponding FTLE from the uncoupled case. For short times, the spectrum is separated between positive and negative FTLEs. For intermediate times, for which the large transient RC are observed, most FTLEs are symmetrically located around the FTLE from the uncoupled case. Some larger positive/negative FTLEs are seen for sites closer to $i=1$ and $i=128$. The number of such larger positive/negative LEs varies for the distinct parametric combinations. Finally, for considerable times for which the RC disappears, we observe distinct behaviors. For the period-1 case related to the point $\blacktriangle$ in Fig. 1, none site *i* assumes the value of the FTLE from the uncoupled case and they are strongly separated, as shown in Fig. 6a by the dark blue triangles. Cases with period-1 and period-2 related respectively to the points $\blacksquare$ and $\blacklozenge$ in Fig. 1, have a similar FTLE spectrum. FTLEs between $i=32$ and $i=96$ are very close to the FTLE from the uncoupled case. For other values of *i*, the FTLEs get apart. In the case of Fig. 6d, all FTLEs are negative and equal to the FTLE from the uncoupled case. This is the only case that is not chaotic after coupling the maps. Furthermore, in all cases the magnitude of the largest negative FTLEs is greater than the magnitude of the largest positive LEs.

## Discussion

Results from Fig. 2a–k demonstrate that collective transient RCs are observed for $N=64,~128,~256,~512$, and 1024 elastically interacting particles. We also demonstrate one case having a coupling-induced collective transient RC through the asymmetric destruction of attractors in Fig. 2j,k. Except for these cases (Fig. 2j,k), the transient RCs approach the RCs from the uncoupled case, plotted by the black curves. In all cases, the transient times and the magnitude of the collective transient RCs increase as *N* increases. For larger couplings strengths, all RCs tend to zero. The large collective transient RCs observed for small couplings are generated when the $p_i$’s values access, for finite times, identical copies of the attractors from the uncoupled case which can be found out of the range $[-2\pi , 2\pi ]$. However, due to the coupling, these attractors become transient. For example, Fig. 6a demonstrates that for times for which the large transient RCs occur, almost all FTLEs are close to the FTLE from the uncoupled case. The same is observed for all other cases. For the parametric combination indicated by $\bullet$ in Fig. 1, in distinction to all other cases, increasing the coupling strength from $\beta =10^{-6}\rightarrow 10^{-4}$ the generated RCs increase in magnitude, as seen by comparing Fig. 2j,k.

The increasing of the collective RC’s transient times, or RC’s lifetime, obeys a power-law $\propto N^{\chi }$, as shown in Fig. 4a (for $\beta =10^{-6}$) and 4b (for $\beta =10^{-4}$). The exponents found are $\chi =1.96$ and $\chi =1.51$, respectively. Thus, we expect to observe $\tau \rightarrow \infty$ as $N\rightarrow \infty$, and that this limit is reached faster for smaller couplings. Interesting to mention is that increasing *N*, the magnitude of the time of the collective transient RCs increases. However, the magnitude of the collective transient RCs is more or less affected by $\beta$, depending on the underline dynamics. For example, compare Fig. 2b,e. The same coupling strength $\beta =10^{-4}$ has a stronger effect on the magnitude of the collective transient RC from Fig. 2e. This is explained using the corresponding FTLE spectrum from Fig. 6a,c. The latter has many positive FTLEs in the transient regime (see the dark-green points) and the LE from the uncoupled case is lesser negative. Thus, the case from Fig. 2e tends to be more unstable, and consequently, the magnitude of the RC is more affected by the coupling when compared to Fig. 2b. This completely agrees with the demonstration that the RC for this parametric combination is easily affected by external noise when compared to the other parameters considered here^45^. Furthermore, this is also the only case for which a collective transient RC’s reversal is observed, from $J\sim -4\pi \rightarrow 2$ for times $10^6\rightarrow 10^8$. Last but not least, the lifetime decreases with a power-law as a function of $\beta$, as seen in Fig. 4c.

Above results explain what has been widely observed in coupled maps systems, that a *“long array of coupled systems may be thought of as a concatenation of small-size sub-systems that evolve almost independently from each other”*^46,47^. However, the implication of such amazing property has never been analysed in the context of collective transient ratchet transport, as done here. The microscopic access to multiple attractors directly affects the macroscopic variable, the RC. The uncoupled case behaves like the thermodynamic limit, at least from the RC point of view. Comparing the exponents $\chi$ and $\mu$ obtained in Fig. 4 with the results above, we can say that the RC reaches faster the values of the uncoupled case when *N* increases than when $\beta$ decreases.

The physical implications of the efficient collective transient ratchet transport for technological applications is noticeable. We mention classical and quantum possibilities for which the collective transport could be of major relevance, like organic electronic ratchets^48^, separating leukocytes from whole blood using the microfluidic ratchet mechanism^49^, tunneling in two-dimensional semiconductors^50^, mesoscopic electronic transport^51^ and polystyrene microspheres immersed in water^52^, to mention a few. Furthermore, the diffusive-like coupling considered here is widespread and can be implemented in some realistic applications, for example, in the coherent transfer in nanoelectromechanical networks^53^, in experiments with coupled electrochemical reactions^54^, in biomolecular motors as in muscles composed of linear structures, which consist of many parts^55^ and in proteins of the kinesin superfamily, where the kinesin direction of motion of the two-headed molecules along microtubules could be reversed by adjusting the architecture of a small domain of the protein named the neck region^56,57^.

## Methods

The time evolution of the relevant physical quantities is obtained by considering *N* elastically coupled Ratchet Maps (RMs)^44,58^ in the form

1 $$\begin{aligned} \mathbf{RM:=}\left\{ \begin{array}{llll} p^{\prime }_{i} =&{} \gamma p_{i} + K [\sin (x_{i}) + a \sin (2 x_{i} + \phi )]+\, F_{\mathrm{coup}}(x_{i-1},x_{i},x_{i+1}), \\ x^{\prime }_{i} =&{} x_{i} + p^{\prime }_{i}, \\ \end{array} \right. \end{aligned}$$

where the first neighbor coupling between the ratchets follows^59,60^

2 $$\begin{aligned} F_{\mathrm{coup}} = \beta (x_{i+1} - 2x_{i} + x_{i-1}), \end{aligned}$$

with *K* being the nonlinearity parameter and $\beta$ the effective coupling strength between the ratchets. Usually, we write $\beta =\epsilon /N$, where $\epsilon$ is the coupling strength between the ratchets and the limit of infinite size is obtained using $N\rightarrow \infty$, which implies that $\beta \rightarrow 0$. The variables $p_i$ is the conjugate momentum of $x_i$ with $i=1,\ldots N$. The one step discrete time evolution of these variables is represented by the prime. In the present work, we used the parameters $a=0.5$ and $\phi =\pi /2$^44,61^, and change $\beta$ and *N*. The above model describes the dynamics of *N* coupled particles moving in an asymmetric potential along the $x_i$ direction with $x_i\in (-\infty ,+\infty )$, while $\gamma _i \in [0,1]$ represents the dissipation of particle *i*. With the recent technological advances, ratchet systems following the above model can be implemented^48–52^.

The *Ratchet Current* (RC) *J* is defined as the double average of the momentum $p_i$, namely

3 $$\begin{aligned} J = \dfrac{1}{N} \displaystyle \sum _{i=1}^N \left[ \dfrac{1}{n} \displaystyle \sum _{j=1}^n p_{i,j}^{\prime } \right] , \end{aligned}$$

where *n* is the total iteration time and *N* the number of RMs. Each one of the *N* maps assumes a different initial condition (IC), and the set of ICs is chosen to be equally distributed inside the intervals $(x_{i}^{\text{ min }},x_i^{\text{ max }})=(p_i^{\text{ min }}, p_i^{\text{ max }})=(-2\pi ,2\pi )$, so that no preferred direction for the entire coupled system is given in the beginning. The effect of the variation of the asymmetry parameter *a* on the RC was studied recently for a single ratchet^62^. While for $a=0$ the RC vanishes, changes of $a\rightarrow -a$ lead to a current reversal, creating the possibility of properly choosing the direction of the RC.

The FTLE spectrum is obtained numerically using the Gram-Schmidt reortonormalization procedure^63,64^. The ICs are equally distributed in the interval $(x_{i}^{\text{ min }},x_i^{\text{ max }})= (p_i^{\text{ min }},p_i^{\text{ max }})=(-2\pi ,2\pi )$, with each RM assuming a different IC. The values for the iteration time *n* used in each simulation are displayed in the panels of Fig. 6.
